# Dupilumab-Induced Psoriasis in a Patient With Chronic Rhinosinusitis With Nasal Polyps: A Case Report

**DOI:** 10.7759/cureus.95219

**Published:** 2025-10-23

**Authors:** Rehab Simsim, Jeffery Cowger, Leigh Sowerby

**Affiliations:** 1 Surgery, Princess Nourah Bint Abdulrahman University, Riyadh, SAU; 2 Dermatology, Advance Medical Group, Ontario, CAN; 3 Rhinology and Anterior Skull Base Surgery, University of Western Ontario, Ontario, CAN

**Keywords:** allergic asthma, atypical rash, chronic rhinosinusitis with nasal polyps, dupilumab, psoriasis

## Abstract

Dupilumab, an IL-4Rα antagonist, has shown substantial efficacy in the treatment of chronic rhinosinusitis with nasal polyps (CRSwNP). However, paradoxical dermatologic side effects, including new-onset psoriasis, have recently been reported. We describe a 42-year-old woman with CRSwNP who developed psoriatic skin lesions after initiating dupilumab, despite no personal or family history of psoriasis. She was started on roflumilast 0.3% topical foam daily, resulting in partial resolution of the psoriatic plaques over the following months. This adverse event may be attributed to an immune shift from a Th2-dominant to a Th1/Th17-driven inflammatory response. Clinicians should remain vigilant for new-onset cutaneous reactions during dupilumab therapy. Early recognition may support timely intervention and modification of therapy.

## Introduction

Chronic rhinosinusitis with nasal polyps (CRSwNP) is a subtype of chronic rhinosinusitis characterized by persistent eosinophilic inflammation and activation of the type 2 immune pathway, particularly involving interleukins IL-4, IL-5, and IL-13. Dupilumab is a fully human monoclonal antibody that targets IL-4Rα, thereby inhibiting IL-4 and IL-13 signaling and suppressing type 2 inflammation. In the landmark SINUS-24 and SINUS-52 trials, dupilumab significantly reduced polyp size, improved nasal airflow, and enhanced quality of life in patients with CRSwNP. Dupilumab is also indicated for the treatment of atopic dermatitis and severe asthma [1].

Although dupilumab is generally well tolerated, rare paradoxical dermatologic side effects have emerged. These include psoriasiform dermatitis and new-onset psoriasis, with several case reports from the atopic dermatitis literature suggesting a causal relationship. These phenomena may be due to an immunologic rebound from suppressed Th2 activity to unopposed Th1/Th17-mediated inflammation. We report a rare case of dupilumab-induced psoriasis in a patient with CRSwNP and no personal or familial history of any dermatologic conditions [2,3].

## Case presentation

A 42-year-old female with a long-standing history of CRSwNP, refractory to two prior endoscopic sinus surgeries and repeated oral corticosteroid courses, the last one being four years ago, presented to our clinic with severe nasal obstruction, anosmia, and postnasal drip. Examination showed grade 1 nasal polyps, both medial and lateral to the middle turbinate, with some eosinophilic mucin present on the right. She has a known case of asthma under control, and an eosinophil count within normal at 0.1.

Computed tomography (CT) of the sinuses revealed diffuse bilateral sinonasal polyposis with opacification of the ethmoid and maxillary sinuses. Her SNOT-22 score was 62, and her sniffin’ sticks identification (SS-I) score was 4, consistent with significant disease burden. Given two previous complete endoscopic sinus surgeries (the most recent 3 years prior) and failure of control with twice-daily budesonide irrigations, she was initiated on dupilumab (300 mg subcutaneously every two weeks). The initial response was favorable, with substantial improvement in nasal obstruction and olfaction (SS-I improved to 8), and the SNOT-22 score dropped to 24 after three months of treatment.

**Figure 1:**
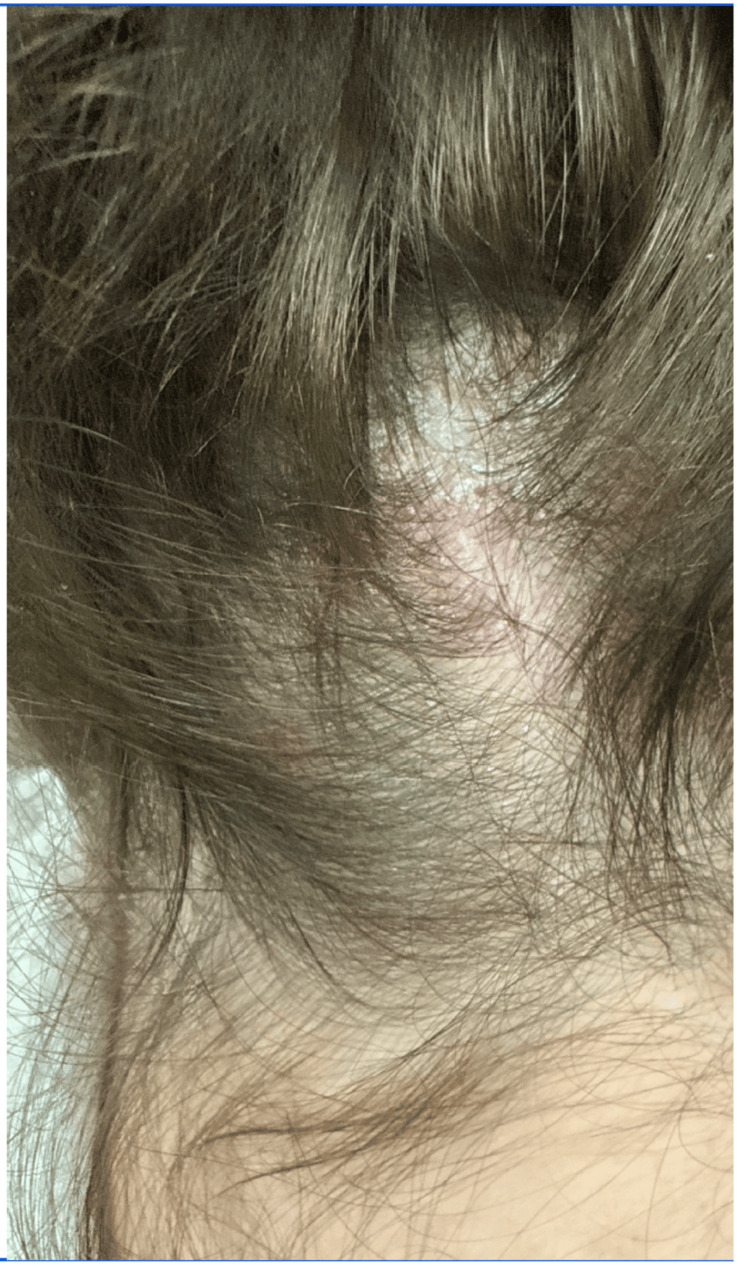
Scalp after treatment withdrawal

**Figure 2:**
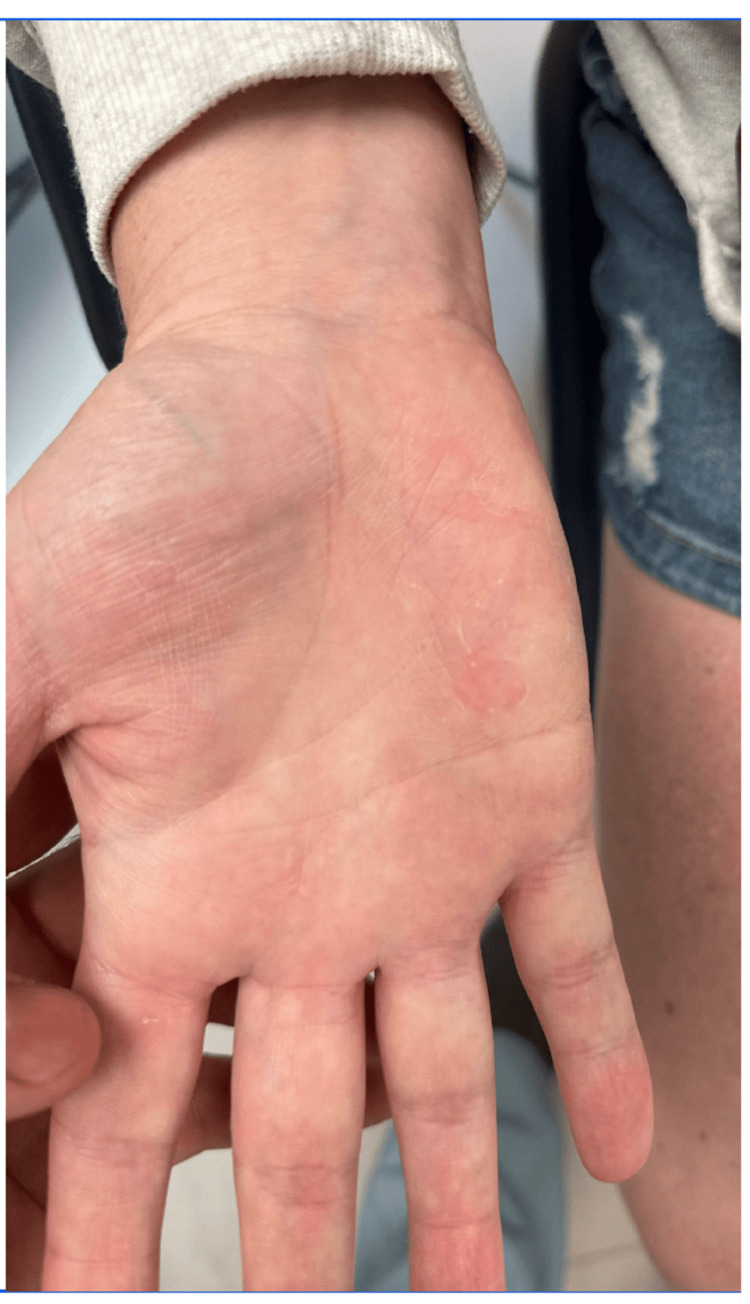
Palms after treatment withdrawal

After 16 weeks of dupilumab therapy, she developed multiple erythematous, scaly plaques over the extensor surfaces of her elbows, knees, and lower back (Figures 1-2). The patient denied any prior history of dermatologic disease and had no family history of psoriasis. Dermatologic consultation confirmed the diagnosis of psoriasis vulgaris. Dupilumab was discontinued, and she was started on roflumilast 0.3% topical foam daily, resulting in partial resolution of the psoriatic plaques over the following months. The complex airway multidisciplinary team later offered switching to an anti-IL-5 monoclonal antibody (mepolizumab) for continued CRSwNP control, but the patient declined further monoclonal therapy.

## Discussion

Dupilumab is widely used for type 2 inflammatory diseases, including CRSwNP. However, paradoxical immune reactions, such as new-onset psoriasis, have been reported in up to 2-3% of patients receiving dupilumab for atopic conditions. The proposed mechanism involves a shift in immune balance by suppressing the Th2 pathway via IL-4/IL-13 blockade; dupilumab may unmask latent Th1/Th17-driven conditions, such as psoriasis. This theory is supported by similar findings in patients treated for atopic dermatitis and asthma who later developed psoriasiform eruptions [4,5].

The majority of side effects from dupilumab are mild in nature. The most common side effects linked to dupilumab include conjunctivitis, reactions at the injection site, nasopharyngitis, and headaches. Additionally, the literature has reported some rare side effects associated with dupilumab. In 2018, Tracey et al. [6] documented the first case of erythrodermic psoriasis associated with dupilumab. Since then, numerous cases of psoriasis and psoriasiform reactions tied to dupilumab have emerged in recent years, revealing a delayed side effect that was not identified during the phase II or phase III trials of dupilumab.

Our case aligns with other reports of psoriatic reactions following dupilumab use in patients without a prior history of psoriasis. Given the chronic nature of both CRSwNP and psoriasis, this raises concerns regarding long-term immunologic effects and the importance of early identification of paradoxical responses. Alternatives such as anti-IL-5 or anti-IgE therapies may be appropriate for these patients, particularly when continued control of CRSwNP is necessary after dupilumab discontinuation [4].

The safety of dupilumab when used alongside other monoclonal antibodies is a significant consideration. Additional studies have reported case series on the use of dupilumab in combination with other biologics (including guselkumab, secukinumab, adalimumab, benralizumab, and omalizumab), showing either no adverse reactions or only mild ones over a monitoring period of 2 to 22 months. Nonetheless, it is essential to conduct long-term follow-ups to assess safety [5].

## Conclusions

This case highlights a rare but important adverse effect of dupilumab therapy: new-onset psoriasis in a patient treated for CRSwNP. This side effect is not well known in the rhinology literature. Clinicians should be aware of this paradoxical reaction and collaborate with dermatologists when new skin lesions develop. Careful monitoring and individualized treatment planning are crucial when managing complex inflammatory diseases treated with biologics.
